# Notes and News

**Published:** 1902-01-18

**Authors:** 


### NOTES AND NEWS.

MR. E. SPEYER, of Speyer Brothers, has contributed £25,000 to the Coronation gift in connection with King Edward's Hospital Fund.

THE number of medical students at the University of Edinburgh maintains its tendency to increase, though the average total is slightly lower this session.

A NEW Isolation Hospital has been erected at Southgate by the Urban District Council. It has been built at a cost of £14,000. Major W. Brown, J.P., performed the opening ceremony.

SIR DONALD CURRIE has sent £1,000 to Mrs. Pirrie for the Royal Victoria Hospital at Belfast. The hospital has good friends. One firm, that of Messrs. Allingham and Co., have contributed 2½ per cent. upon all their sales during December.

THE accounts of the Metropolitan Hospital Saturday Fund for the past year have now been closed. The total receipts were £21,452, an increase of £1,265 on the collection of the previous year, upon which the Fund is much to be congratulated.

EIGHT hundred pounds has been subscribed towards a memorial to the late Professor Hughes, of King's College, and formerly of Cardiff, and to this Mrs. Hughes has added £1,000. The memorial is to take the form of a medal for anatomy at the Cardiff Medical School, and the endowment of an Anatomical Museum at the College. On leaving Cardiff Professor Hughes presented an anatomical collection to the College, and the Museum will now be maintained by means of the Alfred Hughes memorial.

A BAZAAR, in aid of the Hospital for Sick Children, Great Ormond Street, will be held about the time of the Coronation, under the immediate patronage of Her Majesty the Queen, T.R.H. the Prince and Princess of Wales, H.R.H. the Princess Louise, Duchess of Fife and the Duke of Fife, and T.R.H. the Prince and Princess Charles of Denmark. Her Majesty the Queen will be present at the opening of the bazaar, unless unavoidably prevented. Various sites have been offered, or suggested, but it is probable that the bazaar will be held in the garden attached to the hospital itself.

MR. E. OWEN, F.R.C.S., has been appointed consulting surgeon and surgeon-in-chief at the French Hospital, in the place of the late Sir William MacCormac.

THE Admiralty have ordered a gun-boat to be fitted up as a temporary infectious hospital for the navy, in view of the many small-pox cases which have occurred at Chatham. The vessel will be moored in an isolated part of the river Medway.

THE most vigorous protests are being put forward against the retention of the small-pox hospital ships opposite Purfleet. The Asylums Board have promised to disuse the ships as soon as the hospital at Joyce Green is ready, but dissatisfaction is not abated, because two months must elapse before this can be effected.

IN all new countries the value of child life is held very high, and it is not surprising that throughout Australia excellent hygienic and educational measure are being adopted. In New South Wales the separate home system for the care and education of the crippled and feeble-minded finds favour, and individual care in scattered dwellings for orphans and afflicted children is mostly resorted to in Victoria. The care for the child commences with the care of the mother before its birth, and throughout the educational period the State finds means of intervening where conditions are not satisfactory. The offences of childhood are treated in a separate court in South Australia.

THE report of the Statistical Committee of the Asylums Board now published will be read with the greatest interest by many of the public as well as by the medical faculty. Reliable statistics have been eagerly looked for for some time. Briefly in the case of vaccination the results of observations are as follows:—The mortality rate per cent. of vaccinated cases has been 14·21, of doubtful cases 65·08, and of unvaccinated 50·52. Amongst the doubtful cases there were probably many who at any rate had not been revaccinated, and this conclusion will be useful to those who are pondering the question "to be or not to be" at the present time. And of these there are a great many.

THE immense improvement of the health of the Cuban community since it has been under American control, is a testimony to the advantage accruing from modern sanitary knowledge and methods. The Island has been one of hygienic ill-fame for centuries, but vigorous methods have not only minimised the effects of yellow fever, but have greatly reduced the prevalence of malaria and the mortality generally. Such striking results will encourage those who have commenced a crusade against disease in the tropics. Some reports, however, are very depressing, as in the case of a recent letter from Sierra Leone which denies any progress in rendering the locality more healthy. The extermination of the mosquito and the consequent banishment of malaria is regarded by the writer as but a small matter where the natural conditions are so inimical to health. It is hard to remain sanguine in the face of such opinions, but that is what those who undertake a crusade of hygiene must do.

